# Blockade of mTORC1 via Rapamycin Suppresses 27-Hydroxycholestrol-Induced Inflammatory Responses

**DOI:** 10.3390/ijms251910381

**Published:** 2024-09-26

**Authors:** Nakyung Kang, Jaesung Kim, Munju Kwon, Yonghae Son, Seong-Kug Eo, Ninib Baryawno, Byoung Soo Kim, Sik Yoon, Sae-Ock Oh, Dongjun Lee, Koanhoi Kim

**Affiliations:** 1 Department of Pharmacology, School of Medicine, Pusan National University, Yangsan 50612, Republic of Korea; ka776@naver.com (N.K.); rlawotjd1029@naver.com (J.K.); squall0211@hanmail.net (Y.S.); 2 Department of Convergence Medicine, School of Medicine, Pusan National University, Yangsan 50612, Republic of Korea; gmj0226@naver.com; 3 College of Veterinary Medicine and Bio-Safety Research Institute, Jeonbuk National University, Iksan 54596, Republic of Korea; vetvirus@chonbuk.ac.kr; 4 Childhood Cancer Research Unit, Department of Women’s and Children’s Health, Karolinska Institute, 17177 Stockholm, Sweden; n.baryawno@ki.se; 5 School of Biomedical Convergence Engineering, Pusan National University, Yangsan 50612, Republic of Korea; bskim7@pusan.ac.kr; 6 Department of Anatomy, School of Medicine, Pusan National University, Yangsan 50612, Republic of Korea; sikyoon@pusan.ac.kr (S.Y.); hedgehog@pusan.ac.kr (S.-O.O.); 7 Transplantation Research Center, Research Institute for Convergence of Biomedical Science and Technology, Pusan National University Yangsan Hospital, Yangsan 50612, Republic of Korea

**Keywords:** 27-hydroxycholesterol, mTOR, rapamycin, inflammation, monocytic cells

## Abstract

Atherosclerosis is characterized by the deposition and accumulation of extracellular cholesterol and inflammatory cells in the arterial blood vessel walls, and 27-hydroxycholesterol (27OHChol) is the most abundant cholesterol metabolite. 27OHChol is an oxysterol that induces immune responses, including immune cell activation and chemokine secretion, although the underlying mechanisms are not fully understood. In this study, we investigated the roles of the mechanistic target of rapamycin (mTOR) in 27HChol-induced inflammation using rapamycin. Treating monocytic cells with rapamycin effectively reduced the expression of CCL2 and CD14, which was involved with the increased immune response by 27OHChol. Rapamycin also suppressed the phosphorylation of S6 and 4EBP1, which are downstream of mTORC1. Additionally, it also alleviates the increase in differentiation markers into macrophage. These results suggest that 27OHChol induces inflammation by activating the mTORC1 signaling pathway, and rapamycin may be useful for the treatment of atherosclerosis-related inflammation involving 27OHchol.

## 1. Introduction

Atherosclerosis is characterized by the deposition and accumulation of extracellular cholesterol and inflammatory cells within the arterial vessel wall, leading to the formation of plaques [1]. This pathology is marked by a persistent inflammatory response, with inflammatory markers appearing concomitantly with the initial lipid accumulation in the arterial wall [1,2]. Oxysterols, oxygenated derivatives of cholesterol, are biologically active molecules produced through enzymatic and nonenzymatic oxidation [3]. These molecules are involved in numerous physiological and pathological processes, including immune modulation, inflammation, and cytotoxicity [4,5,6]. In atherosclerosis, oxysterols are abundantly present in atherosclerotic plaques, where they actively contribute to plaque development [6,7,8]. Among these metabolites, 27-hydroxycholesterol (27OHChol) is the most prevalent cholesterol metabolite in atherosclerosis and known to elicit inflammatory responses, including the activation of immune cells and the secretion of chemokines [9].

The inflammatory milieu in atherosclerosis is complex and involves multiple signaling molecules and pathways. 27OHChol has been shown to upregulate the expression of *CCL2*, also known as *MCP-1*, and other inflammation-related genes in cells of the monocyte–macrophage lineage [8]. CCL2, a member of the CC chemokine family, binds to the CCR2 receptor and is a critical modulator of inflammation. It plays a significant role in recruiting monocytes, natural killer cells, and T-cell subpopulations to sites of inflammation, contributing to the progression of various diseases, including atherosclerosis [10]. Increased expression of CCL2 has been observed in human atherosclerotic plaques, vascular endothelial cells, and smooth muscle cells exposed to minimally modified lipids [11,12,13]. Macrophages, the key players in the immune response, are major sources of extracellular proteases and cytokines that regulate extracellular matrix remodeling, inflammatory cell recruitment and activation, and vascular smooth muscle cell proliferation and apoptosis [14,15,16,17]. Overall, the activity of CCL2, secreted by inflammatory macrophages, is crucial for the recruitment of monocytic cells into atherosclerotic plaques, exacerbating the inflammatory response and plaque development [18].

The PI3K/AKT/mechanistic target of the rapamycin (mTOR) pathway is pivotal in regulating cell growth, proliferation, survival, and metabolic homeostasis [19,20]. mTOR, a central component of this pathway, exists in two distinct complexes: mTORC1 and mTORC2. Among these, mTORC1 is particularly important for cell growth regulation, as it integrates signals from cellular energy status, amino acid availability, and external growth factors [21,22,23,24]. Consequently, mTORC1 mediates its effects through the phosphorylation of key proteins such as S6K1 and 4EBP1, which are involved in protein synthesis [25,26]. In this context, rapamycin, a potent mTOR inhibitor, specifically inhibits mTORC1 by blocking the phosphorylation of several translational regulators, including S6K1 and 4EBP1 [27,28]. Recent studies have further elucidated the role of mTORC1 in inflammatory processes [29,30,31]. The inhibition of mTORC1 by rapamycin has been shown to reduce the production of proinflammatory cytokines and chemokines, indicating its potential as a therapeutic target in inflammatory diseases [32,33,34]. Furthermore, in the context of atherosclerosis, rapamycin’s ability to inhibit mTORC1 may help mitigate the inflammation induced by 27OHChol, thus preventing plaque progression and stabilizing existing plaques [35].

In this study, we investigated the role of mTORC1 in regulating the inflammatory response induced by 27OHChol in human monocyte-derived THP-1 cells. We aimed to understand the effects of 27OHChol on the PI3K/AKT/mTOR signaling pathway and determine the impact of rapamycin on this pathway and the subsequent inflammatory response. Our findings indicated that treatment with 27OHChol enhanced the phosphorylation of mTORC1 downstream targets, such as the ribosomal proteins S6 and 4EBP1, in THP-1 cells. Rapamycin treatment effectively inhibited mTORC1 signaling and suppressed the 27OHChol-induced inflammatory response. These results suggest that mTORC1 plays a critical role in mediating 27OHChol-induced inflammation and that rapamycin may offer therapeutic potential in managing atherosclerosis by targeting this pathway.

## 2. Results

### 2.1. Effects of Rapamycin on 27OHChol-Induced Inflammatory Responses in THP-1 Cells

**Figure 1:**
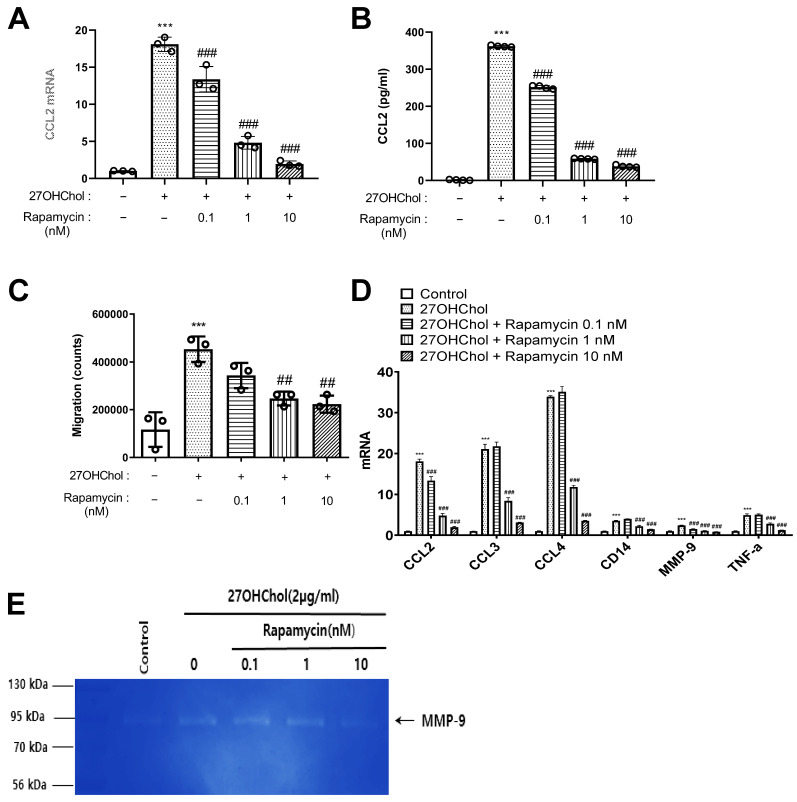
Expression of inflammatory molecules and MMP-9 following treatment with rapamycin. THP-1 cells were serum-starved for 24 h. The cells were treated with 27OHChol (2 μg/mL) in the presence of the indicated amount of rapamycin for 48 h. (**A**) *CCL2* transcript levels were analyzed by real-time PCR. Data are expressed as mean ± SD (*n* = 3 replicates/group). *** *p* < 0.001 vs. control; ### *p* < 0.001 vs. 27OHChol. (**B**) The amount of CCL2 secreted into the medium was measured by ELISA. Data are expressed as mean ± SD (*n* = 3 replicates/group). *** *p* < 0.001 vs. control; ### *p* < 0.001 vs. 27OHChol. (**C**) The migration of monocytic cells was determined using chemotaxis assays. Data are expressed as mean ± SD (*n* = 3 replicates/group). *** *p* < 0.001 vs. control; ## *p* < 0.01 vs. 27OHChol. (**D**) Transcript levels of *CCL2*, *CCL3*, *CCL4*, *CD14*, *MMP-9*, and *TNF-α* were assessed by real-time PCR. Data are expressed as mean ± SD (*n* = 3 replicates/group). *** *p* < 0.001 vs. control; ### *p* < 0.001 vs. 27OHChol. (**E**) Culture media were isolated, and the activity of MMP-9 secreted by the cells was assessed by gelatin zymography. PCR, polymerase chain reaction; SD, standard deviation; 27OHChol, 27-hydroxycholesterol.

To determine whether rapamycin was involved in the inflammatory response induced by 27OHChol, we investigated the expression of inflammatory chemokines in THP-1 cells. Treatment with 27OHChol significantly increased the transcript level of CCL2 by 18.08-fold. However, when rapamycin was co-treated at 0.1, 1, and 10 nM, the increase in the transcript level was concentration-dependently inhibited by 13.36-, 4.8-, and 1.94-fold, respectively (Figure 1A). The secretion of the CCL2 protein was also inhibited by rapamycin treatment, which was consistent with the gene expression pattern (Figure 1B). Next, the effect of rapamycin on cell migration induced by 27OHChol was determined (Figure 1C). We conducted a chemotaxis assay using conditioned media from cells cultured under the experimental conditions described above. The conditioned medium from cells stimulated with 27OHChol enhanced monocyte migration, whereas the medium from cells co-treated with rapamycin showed relatively decreased cell migration. Rapamycin also affected the expression of other inflammatory genes, including CCL3, CCL4, CD14, MMP-9, and TNF-α, all of which exhibited increased transcription levels following 27OHChol treatment (Figure 1D). The assessment of secreted matrix metalloproteinase-9 (MMP-9) activity via gelatin zymography showed that the activity increased by 27OHChol was decreased by 10 nM rapamycin treatment, supporting that rapamycin inhibits the inflammatory response increased by 27OHChol (Figure 1E).

### 2.2. Effects of Rapamycin on the Downstream Proteins of the mTORC1 Signaling Pathway in THP-1 Cells

**Figure 2:**
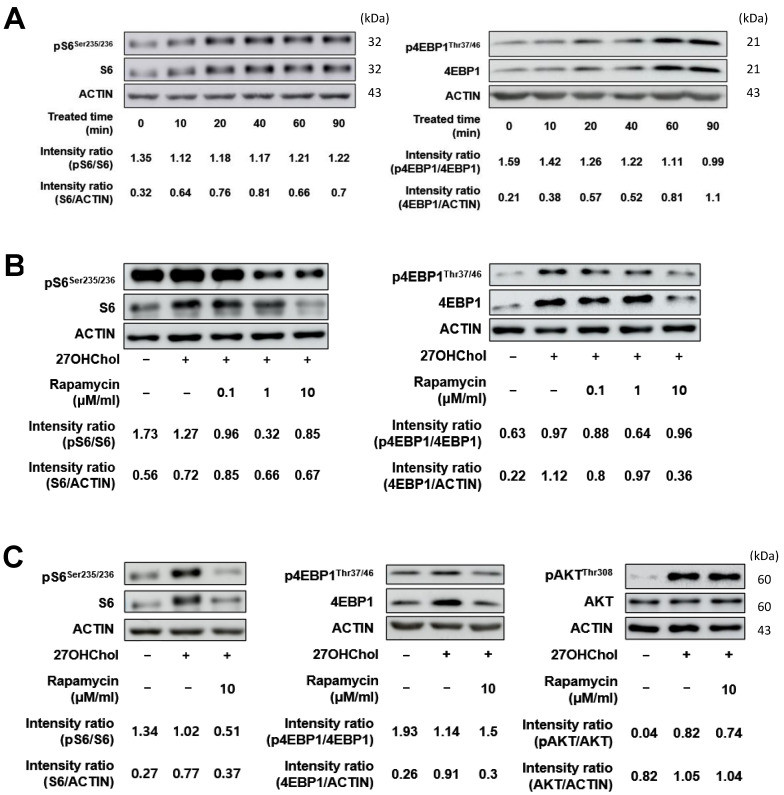
Immunoblots of upstream and downstream proteins of mTORC1 signaling after treatment with 27OHChol. (**A**) THP-1 cells were serum-starved for 24 h. The serum-starved cells were stimulated with 27OHChol (2 μg/mL) for 0, 10, 20, 40, 60, and 90 min. Western blot analysis was used to detect S6, 4EBP1, and their phosphorylated forms. (**B**) The cells were treated with 27OHChol (2 μg/mL) in the presence of rapamycin (10 nM) for 40 min. The indicated proteins were detected by immunoblotting. (**C**) The serum-starved cells were treated with 27OHChol (2 μg/mL) in the presence of varying concentrations of rapamycin for 40 min. Phosphorylated and total levels of S6 and 4EBP1 were detected by immunoblotting. mTORC1, mechanistic target of rapamycin complex 1; 27OHChol, 27-hydroxycholesterol.

Rapamycin is a potent inhibitor of mTOR, a key regulator of cell growth, proliferation, and metabolism, and is known for its therapeutic potential in diseases such as cancer, diabetes, obesity, neurological disorders, and genetic conditions [36]. Recent studies have demonstrated that rapamycin is primarily an antagonist that effectively inhibits mTORC1 [37,38,39] and can inhibit mTORC2 activity in certain cell types [40,41]. To determine whether rapamycin affects the mTORC1 signaling pathway following 27OHChol treatment, we analyzed the expression of phosphorylated S6 (pS6) and phosphorylated 4EBP1 (p4EBP1). The levels of pS6 increased in the presence of 27OHChol, and the increase peaked 40 min after treatment (Figure 2A). p4EBP1 also increased in the same pattern, peaking at 60 min post-treatment. Rapamycin treatment concentration-dependently attenuated this increase in phosphorylation (Figure 2B), and accordingly, the concentration of rapamycin treatment was set to 10 nM in subsequent experiments. In the context of AKT, 27OHChol treatment increased AKT phosphorylation, while rapamycin treatment did not reduce it (Figure 2C). This is probably because AKT is a downstream of both mTOR1 and mTORC2 [42]. These results collectively suggest that the inflammatory response increased by 27OHChol was mitigated by rapamycin treatment through the mTORC1 signaling pathway.

### 2.3. Effects of Rapamycin on Expression of CD14 and on Super-Induction of CCL2

**Figure 3:**
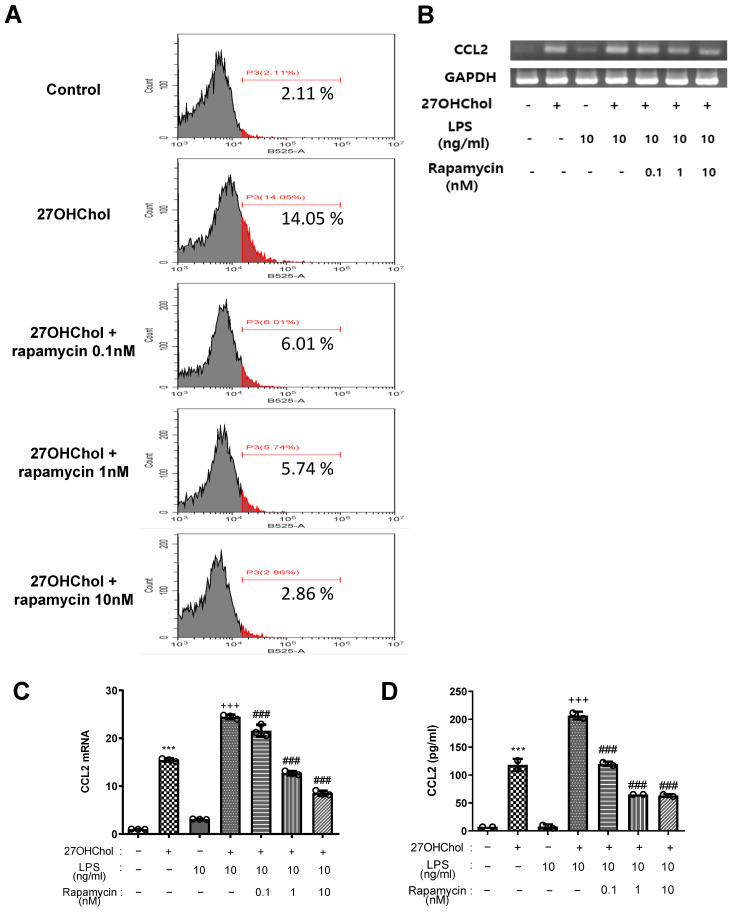
Inhibition of 27HChol-induced CD14 expression and inflammatory response by rapamycin in THP-1 cells. Serum-starved THP-1 cells were cultured with 27OHChol in the presence of the indicated amount of rapamycin for 48 h. (**A**) THP-1 cells were immunostained for surface CD14 and analyzed using flow cytometry. THP-1 cells (2.5 × 10^5^ cells/mL) were serum-starved and incubated for 24 h with 27OHChol in the absence or presence of varying amounts of rapamycin, followed by stimulation for 9 h with or without LPS. (**B**) *CCL2* transcripts were amplified by RT-PCR, and (**C**) levels of *CCL2* transcripts were analyzed by real-time PCR. Data are expressed as mean ± SD (*n* = 3 replicates for each group). *** *p* < 0.0001 vs. control; ### *p* < 0.0001 vs. 27OHChol; +++ *p* < 0.0001 vs. 27OHChol plus LPS. (**D**) Culture media were isolated, and the levels of CCL2 in the media were measured by ELISA. Data are expressed as mean ± SD (*n* = 2 replicates for each group). *** *p* < 0.0001 vs. control; ### *p* < 0.0001 vs. 27OHChol; +++ *p* < 0.0001 vs. 27OHChol plus LPS. RT-PCR, reverse transcriptase polymerase chain reaction; SD, standard deviation; 27OHChol, 27-hydroxycholesterol.

CD14 is mainly expressed in monocytic cells, such as macrophages [43,44], and plays a crucial role in the innate immune system. It binds to bacterial lipopolysaccharides (LPSs), which are components of the outer membrane of Gram-negative bacteria, and initiates a signaling cascade that activates the immune response [45,46,47]. Thus, increased CD14 expression in the cell membrane indicated that the immune response of monocytic cells was further activated. When THP-1 cells were stimulated with 27HChol, CD14 expression significantly increased by more than six-fold, and the expression decreased depending on the concentration of rapamycin (Figure 3A). Subsequently, LPS was administered to assess the changes in CCL2 as an inflammatory response related to CD14 interaction. Reverse transcriptase polymerase chain reaction (RT-PCR) analysis of total ribonucleic acid (RNA) levels (Figure 3B) and quantitative RT-PCR analysis of messenger RNA (mRNA) levels (Figure 3C) demonstrated that *CCL2* transcripts were significantly increased in the presence of 27OHChol. However, upon LPS treatment, *CCL2* expression decreased in a concentration-dependent manner, and CCL2 protein levels secreted into the medium exhibited a similar trend (Figure 3D). These findings suggested that 27HChol enhances the expression of CD14 in monocytic cells and amplifies the immune response to LPS, whereas rapamycin inhibits the immune response resulting from their interaction. 

### 2.4. Effects of Rapamycin on Maturation and Functional Changes in Monocytic Cells Induced by 27HChol

**Figure 4:**
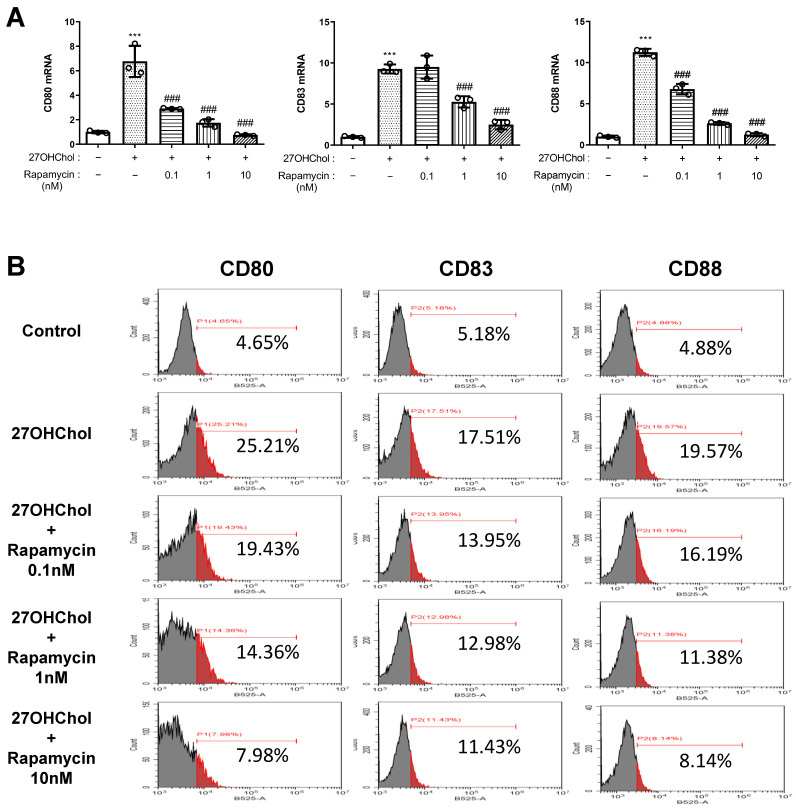
Effects of rapamycin on the maturation of monocytic cells induced by 27HChol. Serum-starved THP-1 cells were cultured with 27OHChol in the presence of the indicated amounts of rapamycin for 48 h. (**A**) Transcription of *CD80*, *CD83*, and *CD88* was assessed by real-time PCR. Data are expressed as mean ± SD (*n* = 3 replicates for each group). *** *p* < 0.0001 vs. control; ### *p* < 0.0001 vs. 27OHChol. (**B**) Cells were immunostained with antibodies against CD80, CD83, and CD88 and analyzed by flow cytometry. PCR, polymerase chain reaction; SD, standard deviation; 27OHChol, 27-hydroxycholesterol.

Monocytes undergo maturation and differentiation to assume more potent and specialized roles within the immune system [48]. Thus, we investigated the changes in the maturation and differentiation of monocytes and their functions induced by 27OHChol and examined the expression changes of representative differentiation markers of myeloid dendritic cells (mDCs), namely CD80, CD83, and CD88 [49,50]. Both mRNA transcription and surface expression of these markers were significantly increased by 27OHChol and decreased in a concentration-dependent manner with rapamycin treatment (Figure 4A,B). This finding suggests that 27HChol promotes the differentiation into DCs with heightened immune responses. Additionally, we explored whether 27HChol induced functional changes in monocytic cells.

**Figure 5:**
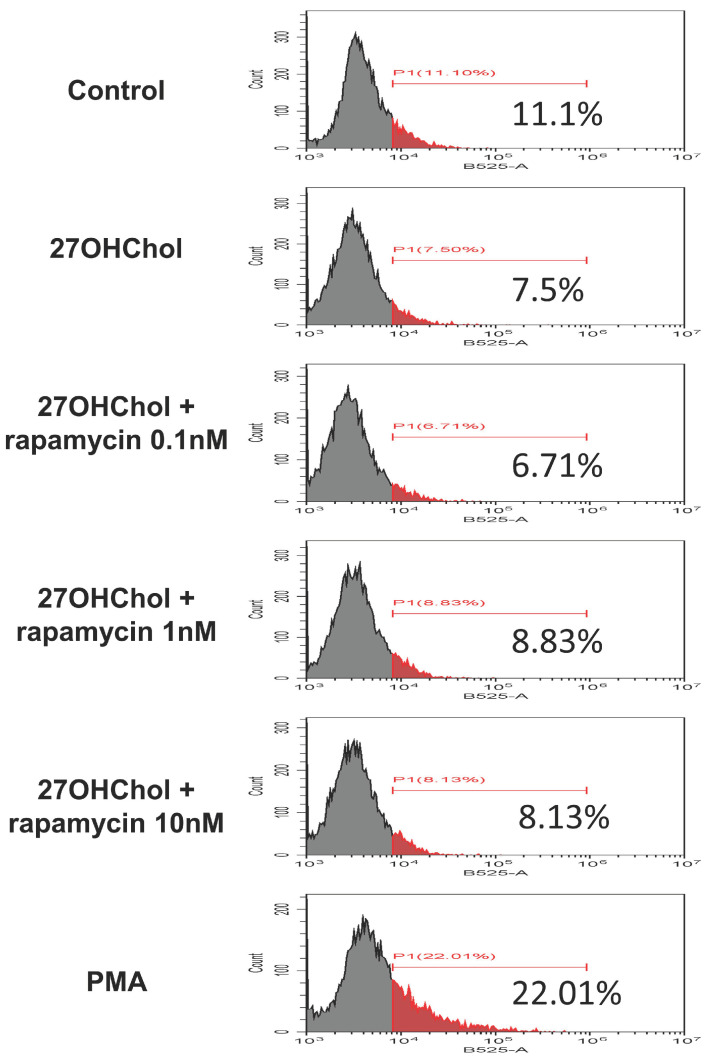
Effects of rapamycin on functional changes in monocytic cells induced by 27Hchol. Serum-starved THP-1 cells (2.5 × 10^5^ cells/mL) were cultured with 27OHChol in the presence of the indicated amounts of rapamycin for 48 h or 0.25 μM of phorbol myristate acetate (PMA) for 24 h. The fluorescence of the cells was analyzed by flow cytometry after incubation with 0.5 mg/mL FITC-conjugated dextran for 30 min. 27OHChol, 27-hydroxycholesterol; FITC, fluorescein isothiocyanate.

Endocytosis is a critical immune function that facilitates phagocytosis, enabling the detection and elimination of antigens or the removal of dead cells at sites of inflammation [51]. Our results indicate that 27OHChol treatment did not show a significant alteration in endocytosis in monocytes, and rapamycin treatment also did not seem to affect this function (Figure 5). Overall, our findings suggest that while 27OHChol induces the maturation and differentiation of monocytic cells into DCs, rapamycin treatment can inhibit this maturation without compromising the fundamental immune function of endocytosis.

## 3. Discussion

Previous studies have demonstrated that 27OHChol induces abnormal inflammatory responses and affects disease progression in atherosclerotic plaques [52,53]. However, the underlying mechanisms are still not well understood. In this study, we aimed to elucidate these mechanisms by investigating the role of 27OHChol-induced inflammation in monocytes, focusing on the mTORC1 signaling pathway. 

Our results demonstrate that 27OHChol significantly upregulates the expression of CCL2, a key chemokine involved in monocyte recruitment and a prominent inflammatory marker in atherosclerotic plaques [12,13]. However, the considerable increase in CCL2 transcript levels and protein expression following 27OHChol treatment was attenuated by rapamycin in a concentration-dependent manner. This suggests that rapamycin alters the inflammatory signaling pathway by inhibiting CCL2 secretion, indicating a significant role of mTORC1 signaling in this process. Additionally, the chemotaxis assay revealed that the enhanced monocyte migration treated with 27OHChol was reduced when co-administered with rapamycin. This implies that rapamycin not only reduces CCL2 levels in monocytes but also inhibits its extracellular secretion, thereby affecting CCL2 which mediates the recruitment of monocytes. Furthermore, rapamycin also inhibited the activity of MMP-9, which is involved in extracellular matrix remodeling and plaque instability. This finding highlights the role of rapamycin in alleviating the inflammatory and tissue remodeling processes in atherosclerotic plaques.

When exploring mTORC1 signaling, we observed that 27OHChol treatment increased the phosphorylation of the downstream targets S6 and 4E-BP1 [28]. Rapamycin effectively reversed this effect, confirming that 27OHChol activates mTORC1 signaling, which can be specifically inhibited by rapamycin. Notably, rapamycin did not affect AKT phosphorylation, which was increased by 27OHChol. This indicates that its inhibitory effects were specific to mTORC1 rather than mTORC2.

In addition to its effects on CCL2 and cell migration, rapamycin can modulate the expression of CD14, a marker of monocyte activation [45,46]. The increase in CD14 expression induced by 27OHChol was downregulated by rapamycin, suggesting that rapamycin modulates monocyte activation. The reduction in CCL2 expression in response to LPS in the presence of rapamycin further supports the hypothesis that rapamycin inhibits the inflammatory response initiated by 27OHChol. Our examination of monocyte maturation and functional changes revealed that 27OHChol promoted monocyte differentiation into macrophages, as confirmed by increased expression of the differentiation markers CD80, CD83, and CD88 [49,50]. This promotion could accelerate atherosclerotic plaque formation and progression due to enhanced inflammatory responses and lipid accumulation in macrophages. Nevertheless, rapamycin treatment may alleviate pro-inflammatory effects by inhibiting the maturation of monocytes into macrophages while preserving their fundamental endocytic function.

In conclusion, our findings highlight the critical role of mTORC1 in mediating the inflammatory responses induced by 27OHChol. The ability of rapamycin to inhibit mTORC1 signaling and subsequently reduce CCL2 production, cell migration, and dendritic cell maturation suggests its potential as a therapeutic agent for managing atherosclerosis. By targeting mTORC1, rapamycin may help alleviate chronic inflammation associated with atherosclerosis and stabilize atherosclerotic plaques, offering a promising strategy for therapeutic interventions in this prevalent vascular disease.

## 4. Materials and Methods

### 4.1. Cell Culture and Treatment

The human acute monocytic leukemia THP-1 cell line was purchased from the American Type Culture Collection (ATCC; Manassas, VA, USA). THP-1 cells were cultured in RPMI medium 1640 supplemented with 10% fetal bovine serum (FBS) and 1% penicillin–streptomycin in a humidified atmosphere of 5% CO_2_ at 37 °C. The cells were passaged every 2–3 d to maintain between one thousand and one million cells per mL in the culture medium. The cells (2.5 × 10^5^ cells /mL) were serum-starved by incubating them for 24 h in RPMI medium 1640 containing 0.1% bovine serum albumin (BSA; GenDEPOT. Katy, TX, USA) and then treating them with 27OHChol (2 μg/mL) in the absence or presence of rapamycin.

### 4.2. Reagents

27OHChol(SML2042) was purchased from Sigma-Aldrich (St. Louis, MO, USA) and dissolved in ethanol. Antibodies against β-actin(sc-47778), anti-mouse IgG-HRP(sc-516102), anti-rabbit IgG-HRP(sc-2357), 4E-BP1(sc-81149), and phosphorylated 4E-BP1(sc-293124) were purchased from Santa Cruz Biotechnology, Inc., (Santa Cruz, CA, USA). Antibodies against S6 Ribosomal protein(2217s), phosphorylated S6 Ribosomal protein(5364s), Akt(pan)(4691S), and p-Akt(S473)(4060S) were purchased from Cell Signaling Technology, Inc., (Danvers, MA, USA).

### 4.3. Reverse Transcription-Polymerase Chain Reaction (RT-PCR)

RNA was extracted using Trizol reagent, followed by the addition of chloroform to induce phase separation. The aqueous phase containing RNA was carefully collected, and RNA was precipitated by adding isopropanol. The RNA pellet was then washed with 70% ethanol to remove impurities, dried briefly, and dissolved in DEPC-treated water. Finally, the RNA concentration and purity were assessed using a NanoDrop spectrophotometer.

Total RNA was reverse-transcribed to produce cDNA for 1 h at 42 °C using Moloney murine leukemia virus reverse transcriptase (Promega, Madison, WI, USA). In the non-quantitative PCR analysis, GAPDH transcripts were amplified as an internal control. The cDNA was denatured at 95 °C for 10 min, followed by 27–30 cycles at 95 °C for 30 s, 55 °C for 30 s, and a 72 °C elongation period for 30 s. PCR products were separated by electrophoresis on 2% agarose gels for 30 min and visualized using ethidium bromide staining. Images were captured using a WSE-5300 PrintGraph CMOS I (Tokyo, Japan) device. The primer sequences are listed in Table 1

### 4.4. Real-Time Polymerase Chain Reaction (Real-Time PCR)

RNA was extracted in the same way performed on RT-PCR. After reverse-transcription of total RNAs for 1 h at 42 °C with Moloney Murine Leukemia Virus reverse transcriptase, transcripts of the genes of interest were assessed by real-time PCR using a LightCyclere 96 Real-Time PCR System (Roche, Germany) [52]. Each 20 μL reaction consisted of 10 μL of SYBR Green Master Mix, 2 μL of forward and reverse primers (10 pM each) for the genes being analyzed, and a cDNA template. The thermal cycling conditions were as follows: 95 °C for 10 min, and 45 cycles at 95 °C for 10 s, 50 °C for 10 s, and an elongation period for 10 s at 72 °C. The relative expression of each gene was calculated relative to the expression of GAPDH. The primer sequences are listed in Table 2.

### 4.5. Enzyme-Linked Immunosorbent Assay (ELISA)

The levels of CCL2 secreted into the culture media were determined using a commercially available enzyme-linked immunosorbent assay kit (Human MCP-1 ELISA kit, BD Biosciences, San Diego, CA, USA) [54,55]. Standards and samples were added in triplicates to individual wells and incubated for 2 h at room temperature. After washing the wells with washing buffer, a polyclonal antibody conjugated to horseradish peroxidase and preservatives was added to each well and incubated for 1 h at room temperature. The wells were then washed with washing buffer, followed by the addition of the substrate solution to each well for color development. After incubation for 30 min in the dark, the reaction was stopped with 50 μL of stop solution (2 N sulfuric acid). Differences in absorbance were measured at 450 nm.

### 4.6. Western Blot Analysis

Proteins were extracted using PRO-PREP protein extraction solution (iNtRON Biotechnology, Songnam, Republic of Korea). The protein concentration in the cell lysates was determined using a bicinchoninic acid assay. To denature the proteins, cell lysates were mixed with 5× SDS loading buffer (10% SDS, 50% glycerol, 5% β-mercaptoethanol, 0.5% bromophenol blue, and 250 mM Tris-HCl, pH 6.8) and heated at 95 °C for 1 min. The denatured proteins were then separated on 11% SDS-PAGE gels and transferred onto nitrocellulose membranes. After blocking with 1% skim milk in Tris-buffered saline (TBS) containing 0.05% Tween-20 for 1 h at room temperature, the membranes were incubated with primary antibodies diluted in the blocking solution (1: 1000, 1: 100) at 4 °C overnight. The membranes were washed thrice with washing buffer (TBS with 0.05% Tween-20) for 10 min each and then incubated for 1 h with HRP peroxidase-conjugated secondary antibodies diluted in the blocking solution (1:8000) at room temperature. After washing thrice with wash buffer for 10 min each, bands were detected using chemiluminescent detection reagents after immunoblotting (Luminata Forte Western HRP Substrate; Millipore Corporation, Billerica, MA, USA). The chemiluminescence was imaged using an Amersham Imager 680 (GE Healthcare, Madison, IL, USA) device.

### 4.7. Chemotaxis Assay

The migration of THP-1 cells was measured using Transwell permeable supports (Costar, Cambridge, MA, USA). Transwell chambers were inserted into wells filled with supernatants containing CCL2 obtained after the exposure of THP-1 cells to 27OHChol, rapamycin, and/or STA9090. Serum-starved THP-1 cells (5 × 10^5^ cells/100 μL) suspended in RPMI medium 1640 containing 0.1% BSA were loaded into the top chamber of 5 μM-pore polycarbonate Transwell inserts. After incubation for 2 h in 5% CO_2_ at 37 °C, the top chamber was removed. Cells were diluted (1:1) in trypan blue solution, and cell viability was measured using a Vi-Cell cell counter (Beckman Coulter, Inc. Brea, CA, USA). Nonviable cells turned blue, whereas viable cells remained unstained.

### 4.8. MMP-9 Gelatinolytic Activity in Cell Supernatants

MMP-9 activity was assessed using gelatin zymography. THP-1 cells were centrifuged, and the supernatants were isolated and concentrated 20-fold using a Vivaspin 2 Centricon (Sartorius, Göttingen, Germany). The concentrated media were electrophoretically separated onto 8% polyacrylamide gel containing 0.15% gelatin (Sigma-Aldrich, St. Louis, MO, USA). After electrophoresis, the gel was washed and activated for 18 h at 37 °C. Following electrophoresis, the gel was stained with 0.2% Coomassie Brilliant Blue R-250 and destained. Images were captured using a WSE-5300 PrintGraph CMOS I (Tokyo, Japan) system.

### 4.9. Flow Cytometric Analysis

THP-1 cells were harvested by centrifugation and incubated for 4 h at 4 °C with fluorescein isothiocyanate-conjugated anti-CD14, CD80, CD83, and CD88. After washing with phosphate-buffered saline (PBS), the cells were resuspended in PBS. Fluorescence was analyzed using a CytoFLEX device and CytExpert 2.5.0.77 software (Beckman Coulter, CA, USA).

### 4.10. Statistical Analysis

All data are expressed as means ± SD. Statistical analysis by one-way ANOVA followed by Dunnett’s multiple comparison tests was performed using PRISM (version 5.0; GraphPad Software Inc., San Diego, CA, USA). In cases where we were asking about the differences within each group, we applied one-way ANOVA. When we were interested in the differences among the different data points within each group, we applied one-way ANOVA with Tukey’s Multiple Comparison Test. The level of significance is represented as follows: * *p* < 0.05, ** *p* < 0.01, and *** *p* < 0.001.

## Figures and Tables

**Table 1:** Primer sequences for non-quantitative PCR.

Primer Sequences for Non-Quantitative PCR	
Primers	Sequences
Human *GAPDH*	Forward 5′-GAGTCAACGGATTTGGTCCT-3′
	Reverse 5′-TGTGGTCATGAGTCCTTCCA-3″
Human *CCL2*	Forward 5′-TCTGTGCCTGCTGCTCATAG-3′
	Reverse 5′-CAGATCTCCTTGGCCACAAT-3′

PCR, polymerase chain reaction.

**Table 2:** Primer sequences for real-time PCR.

Primer Sequences for Real-Time PCR	
Primers	Sequences
Human *GAPDH*	Forward 5′-GAAGGTGAAGGTCGGAGT-3′
	Reverse 5′-GAAGATGGTGATGGGATTTC-3′
Human *CCL2*	Forward 5′-CAGCCAGATGCAATCAATGCC-3′
	Reverse 5′-TGGAATCCTGAACCCACTTCT-3′
Human *CCL3*	Forward 5′-AGTTCTCTGCATCACTTGCTG-3′
	Reverse 5′-CGGCTTCGCTTGGTTAGGAA-3′
Human *CCL4*	Forward 5′-CTGGGTCCAGGAGTACGTGT-3′
	Reverse 5′-GCGGAGAGGAGTCCTGAGTA-3′
Human *CD14*	Forward 5′-ACGCCAGAACCTTGTGAGC-3′
	Reverse 5′-GCATGGATCTCCACCTCTACTG-3′
Human *MMP-9*	Forward 5′-GCACGACGTCTTCCAGTACC-3′
	Reverse 5′-CAGGATGTCATAGGTCACGTAGC-3′
Human *TNF-α*	Forward 5′-ATGAGCACTGAAAGCATGATCC-3′
	Reverse 5′-GAGGGCTGATTAG AGAGAGGTC-3′
Human *CD80*	Forward 5′-GCAGGGAACATCACCATCCA-3′
	Reverse 5′-TCACGTGGATAACACCTGAACA-3′
Human *CD83*	Forward 5′-TCCTGAGCTGCGCCTACAG-3′
	Reverse 5′-GCAGGGCAAGTCCACATCTT-3′
Human *CD88*	Forward 5′-GTGGTCCGGGAGGAGTACTTT-3′
	Reverse 5′-GCCGTTTGTCGTGGCTGTA-3′

PCR, polymerase chain reaction.

## Data Availability

The data supporting the findings of this study are available upon request from the corresponding author.
